# Increased COX-2 Immunostaining in Urothelial Carcinoma of the Urinary Bladder Is Associated with Invasiveness and Poor Prognosis

**DOI:** 10.1155/2019/5026939

**Published:** 2019-04-21

**Authors:** Basim Al-Maghrabi, Wafaey Gomaa, Mohammed Abdelwahed, Jaudah Al-Maghrabi

**Affiliations:** ^1^Faculty of Medicine, King Abdulaziz University, Jeddah, Saudi Arabia; ^2^Department of Pathology, Faculty of Medicine, King Abdulaziz University, Jeddah, Saudi Arabia; ^3^Department of Pathology, Faculty of Medicine, Minia University, Al-Minia, Egypt; ^4^Department of Pathology, Faculty of Medicine, Al-Azhar University, Cairo, Egypt; ^5^Department of Pathology, Faculty of Medicine, University of Jeddah, Jeddah, Saudi Arabia; ^6^Department of Pathology, King Faisal Specialist Hospital and Research Centre, Jeddah, Saudi Arabia

## Abstract

**Background:**

Urothelial carcinoma of the urinary bladder (UCB) is the commonest bladder tumor. Cyclooxygenase-2 (COX-2) mediates angiogenesis, cell survival/proliferation, and apoptosis. This study investigates the relation of COX-2 immunostaining in UCB to clinicopathological parameters in Saudi Arabia.

**Methods:**

The study population includes 123 UCB and 25 urothelial mucosae adjacent to UCB. UCB samples were collected before any local or systemic therapy. Tissue microarrays were designed and constructed, and TMA blocks were sliced for further immunohistochemical staining. Immunohistochemical staining was done using a mouse anti-human COX-2 monoclonal antibody. A cutoff point of 10% was chosen as the threshold to determine low and high COX-2 immunostaining.

**Results:**

COX-2 immunostaining is higher in UCB than in the adjacent urothelium (*p* = 0.033). High COX-2 immunostaining is associated with high-grade UCB (*p* = 0.013), distant metastasis (*p* = 0.031), lymphovascular invasion (*p* = 0.008), positive muscle invasion (*p* = 0.017), pT2 and above (*p* = 0.003), and high anatomical stages (stage II and above). High COX-2 immunostaining is an independent predictor of higher tumor grade (*p* < 0.001), muscle invasion (*p* = 0.015), advanced pathological T (*p* = 0.014), lymphovascular invasion (*p* = 0.011), and distant metastasis (*p* = 0.039). High COX-2 immunostaining is associated with lower overall survival rate (*p* = 0.019).

**Conclusion:**

COX-2 immunostaining is associated with the invasiveness of UCB which may be used as an independent prognostic marker. COX-2 may be a significant molecule in the initiation and progression of UCB. Molecular and clinical investigations are required to explore the molecular downstream of COX-2 in UCB and effectiveness of COX-2 inhibitors as adjuvant therapy along with traditional chemotherapy.

## 1. Background

Urothelial carcinoma of the urinary bladder (UCB) is the commonest bladder tumor in Western countries [1]. In Saudi Arabia, UCB represents 3.8% of cancers in males [2]. UCB predominantly manifests as a non-muscle-invasive tumor. Low-grade tumors have a good prognosis after transurethral resection while patients with high-grade tumors require intravesical instillation of Bacillus Calmette-Guerin and/or chemotherapy. In 70% of non-muscle-invasive UCB, patients suffer a recurrence following treatment. In 15% of noninvasive UCB, muscle invasion develops. Progression risk is potential in patients with high-grade tumors [3]. The cost of therapy is still high because of the high risk of recurrence and the close lifetime follow-up [4].

Chronic inflammation is thought to increase the risk of UCB [5]. Cyclooxygenase-2 (COX-2) is a prostaglandin endoperoxide synthetase. Proinflammatory cytokines, growth factors, tumor initiators, and other external factors stimulate COX-2 to catalyse prostanoid production [6, 7]. Activation of COX-2 facilitates cellular processes and is involved in tumorigenesis such as angiogenesis, tumor cell proliferation, survival, and apoptosis [8, 9]. Urinary bladder tissue in patients with cystitis or UCB showed increased COX-2 levels as compared to normal urinary bladder tissue [10, 11]. COX-2 is overexpressed in UCB [11–14] and colorectal carcinoma [15].

The objective of this study is to investigate the relation of the COX-2 immunostaining status to various clinicopathological parameters and its value as a predictor of disease outcome in a subset of UCB patients from Saudi Arabia. Up to our knowledge, this is the first study to address this relation in Saudi Arabian UCB patients.

## 2. Methods

### 2.1. Patients

A total of 123 UCB and 25 uninvolved nearby urothelial mucosae adjacent to UCB are included in the current study. All pathological materials were recruited in the Department of Pathology, King Abdulaziz University, Jeddah, Saudi Arabia. UCB samples used in the study were obtained before any intravesical or systemic therapy. Tumors were reviewed regarding the T stage according to the criteria of the Cancer Staging Atlas of the American Joint Committee on Cancer [16], while the grade was revised according to the World Health Organization classification of tumors [17]. The clinicopathological findings are shown in Table 1. The Research Committee of the Biomedical Ethics Unit, Faculty of Medicine, King Abdulaziz University, Jeddah, Saudi Arabia, approved this study. Informed written consents to use their biopsy material in research were obtained from patients included in this study.

### 2.2. Tissue Microarray

The design and construction of tissue microarrays were performed as previously described [18, 19]. Two cores of tissue were selected from each UCB and uninvolved urothelial mucosa and arrayed in recipient paraffin blocks. The automated tissue arrayer (Master 3DHISTECH) was used. Normal placenta tissues were used for orientation of cores in TMA blocks. TMA blocks were sliced into 4-micrometer sections and mounted on positive-charged slides for further immunohistochemical staining.

### 2.3. Immunohistochemistry

Immunohistochemical staining was done using a mouse anti-human COX-2 monoclonal antibody (DakoCytomation Norden A/S, Glostrup, Denmark; dilution 1 : 50). The immunostaining procedure was carried out using an automatic immunostainer (Ventana BenchMark XT, Ventana Inc., Tucson, AZ). Positive controls were used consisting of colorectal carcinomas known to be positive for an anti-COX-2 antibody. Negative controls were treated with tris-buffered saline instead of primary antibody.

### 2.4. Evaluation of COX-2 Immunostaining

A semiquantitative scoring was used by recording the percentage of positive cells for COX-2. In each disk of tissue, the tumor/urothelial cells were counted, and subsequently, the positive cells (cytoplasmic brown staining) were counted. The percentage of COX-2-positive cells was calculated. A cutoff point of 10% was chosen as the threshold. COX-2-negative immunostaining was assigned when less than 10% of the examined tumor/urothelial cells were stained while COX-2-positive immunostaining was considered when equal to or greater than 10% of the examined tumor/urothelial cells were stained [20–23]. Accordingly, we classified COX-2 immunostaining into high and low immunostaining.

### 2.5. Statistical Analysis

Statistical tests were performed in the SPSS® program (NY, USA) version 16. The statistical significance was considered at *p* < 0.05. Testing the difference between two and three variables was calculated by using the Mann-Whitney *U* test, and Kruskal-Wallis tests were used alternatively. Along one variable of data, the variation was tested using the nonparametric chi-squared test. The Kaplan-Meier method with the log-rank (Mantel-Cox) test was used to compare the survival probability. Disease-free survival was calculated as the time from diagnosis to the appearance of recurrent disease (or the date last seen being disease free). Binary logistic regression analysis was utilised to determine the prognostic value of COX-2 immunostaining. The estimated odds ratio (exponential{*B*}) and 95% confidence interval for exp [B] were expressed.

## 3. Results

### 3.1. Pattern of COX-2 Immunostaining

**Figure 1:**
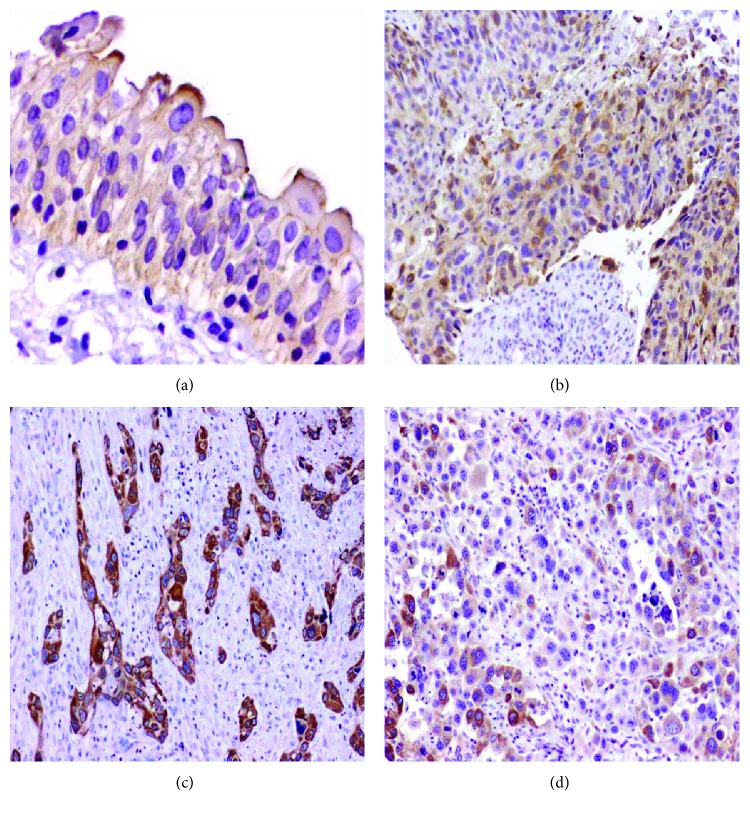
COX-2 immunostaining. (a) A section from a normal urinary bladder mucosa shows low COX-2 immunostaining in the apical portion of umbrella cells (200x). (b) A section from a papillary UCB, (c) a section from an invasive UCB, and (d) a section from high-grade UCB. Cytoplasmic COX-2 immunostaining is higher in invasive and high-grade UCB (100x). Immunohistochemistry was done using an anti-COX-2 antibody, diaminobenzidine as the chromogen, and haematoxylin as a counterstain.

The staining pattern of COX-2 in UCB and the adjacent urothelium is shown in Figure 1. In the adjacent urothelium, COX-2 immunostaining was detected in 22%. COX-2 immunostaining is detected focally in the cytoplasm of umbrella cells of the urothelium adjacent to UCB (Figure 1(a)). Immunostaining was detected in 37.8% of UCB. Diffuse cytoplasmic COX-2 immunostaining in malignant urothelial cells is shown in Figures 1(b)–1(d). The incidence of low COX-2 immunostaining is statistically higher than that of high COX-2 immunostaining in the adjacent urothelium as well as in UCB (*p* ≤ 0.001). However, COX-2 immunostaining is more reported in UCB than in the adjacent urothelium (*p* = 0.033). Data is shown in Table 2.

### 3.2. Correlation of COX-2 Immunostaining with Prognostic Factors of UCB

The distribution of COX-2 immunostaining among clinicopathological parameters is shown in Table 3. High COX-2 immunostaining is statistically associated with tumors with high grade (*p* = 0.013), tumors associated with distant metastasis (*p* = 0.031), and tumors with positive lymphovascular invasion (*p* = 0.008). High COX-2 immunostaining is associated with the invasiveness of UCB. This is shown as statistically positive association with positive muscle invasion (*p* = 0.017), tumors with high pathological T (pT2 and above) (*p* = 0.003), and high anatomical stages (stage II and above). High COX-2 immunostaining has a borderline statistically significant association with positive lymph node metastasis (*p* = 0.08). There is no statistically significant difference in COX-2 immunostaining in relation to age groups, sex, local disease recurrence, and survival status. Regression analysis revealed that high COX-2 immunostaining is an independent predictor of higher tumor grade (*p* < 0.001), muscle invasion (*p* = 0.015), advanced pathological T (*p* = 0.014), lymphovascular invasion (*p* = 0.011), and distant metastasis (*p* = 0.039). Details of regression analysis are shown in Table 4.

### 3.3. Survival Outcome in relation to COX-2 Immunostaining

**Figure 2:**
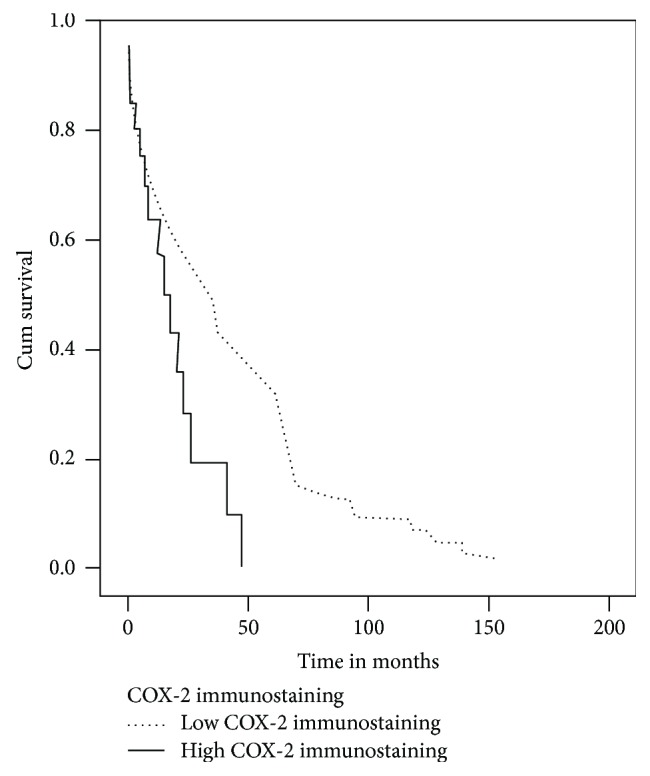
Overall survival curve (Kaplan-Meier) according to COX-2 immunostaining. Low COX-2 immunostaining is associated with better overall survival (log-rank = 5.485, *p* = 0.019).

**Figure 3:**
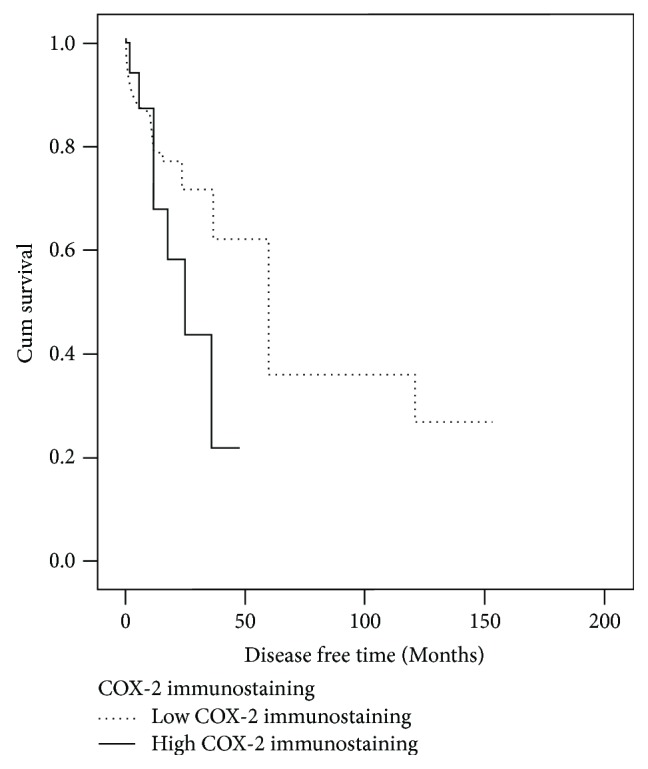
Disease-free survival curve (Kaplan-Meier) according to COX-2 immunostaining. There is no difference in survival probability between low and high COX-2 immunostaining (log-rank = 2.325, *p* = 0.127).

The survival analysis revealed that there is a statistically significant lower overall survival rate in patients with high COX-2 immunostaining than in patients with low COX-2 immunostaining (log-rank (Mantel-Cox) = 5.485 and *p* = 0.019) (Figure 2). Even though disease-free survival is not statistically significant (log-rank (Mantel-Cox) = 2.325, *p* = 0.127), there is a clear trend that patients with tumors with low COX-2 immunostaining have longer disease-free survival and those with tumors with high COX-2 immunostaining have shorter disease-free survival (Figure 3).

## 4. Discussion

COX-2 is not usually expressed in tissues; however, it is induced under certain stimuli including inflammatory cytokines, growth factors, and oncogenes [24]. Increased immunostaining of COX-2 is observed in different tumors including UCB [11, 13]. However, in the normal human urinary bladder epithelium, COX-2 is not expressed [13, 20, 24–26]. On the other hand, other studies demonstrated weak COX-2 immunoreactivity in the urothelium of normal bladder tissue [27]. In the present study, tissues from the morphological normal urothelium adjacent to UCB were examined for COX-2 immunostaining. In the examined specimens, COX-2 showed low immunostaining (<10%) by apical localisation. This observation was previously reported and consistent with our finding [13, 20]. This observation may represent a field effect of carcinogens in urine on urothelial cells surrounding tumor or a paracrine loop via secretion of growth factors or cytokines by neoplastic cells [13]. COX-2 immunostaining was observed in areas of chronic cystitis, squamous metaplasia, and dysplasia [28]. On the other hand, others did not observe COX-2 expression in normal urothelial cells surrounding tumors [29]. This issue is very important and needs to be addressed on large-scale samples as it may be useful to use COX-2 inhibitors as chemopreventive for local tumor recurrence.

COX-2 overexpression may be involved in initiating carcinogenesis [30, 31]. In the present study, high COX-2 immunostaining is statistically higher in UCB than in the adjacent urothelium. High immunostaining was reported in 17.1% of UCB. 10% of immunoreactive cells were used as the cutoff point which was previously used and showed similar results regarding COX-2-positive immunostaining in UCB [22, 24, 32]. However, others reported COX-2 immunostaining in a large number of UCB [13, 21, 33]. Difference in COX-2 immunostaining between normal and tumor tissues may be explained by paracrine effects between normal and tumor tissues. The discrepant results may be due to different technical conditions in COX-2 immunostaining and different interpretation of COX-2 immunostaining.

Most of the well-known prognostic markers of tumors are clinical and pathological parameters including the stage and grade. On the other hand, the assessment of the biological behaviour of tumors is essential to set the appropriate therapeutic modality. So, more reliable prognostic factors are needed. The current approach is to focus on genetic markers [34]. One of the molecules targeted in this process is COX-2 which is considered a risk factor for development and invasion of urinary bladder carcinoma [35]. There are still contradictory results regarding COX-2 expression as an independent prognostic factor of UCB. The association between COX-2 and clinicopathological parameters is a contentious issue. In our study, high COX-2 immunostaining is associated with advanced tumor stages. There are several studies reporting the same association [28, 32, 36]. On the other hand, other studies failed to show association between COX-2 expression and grade in UCB [20, 22, 24, 29, 37, 38].

It was reported that COX-2 helps the invasive ability on malignant cells in vitro and may be involved in UCB carcinogenesis [24, 30, 39]. The results from our study showed that high COX-2 immunostaining is associated with the invasiveness of UCB. This is expressed by positive association with muscle invasion, high pathological T (pT2 and above), and high anatomical stages (stage II and above). Similar previous findings were reported in previous studies [11, 32, 40]. But there are some other studies which failed to prove such association [29, 36]. Also, we reported that high COX-2 immunostaining is statistically associated with tumors with positive lymphovascular invasion similar to that of a previous report [36]. These findings may support the potential role of COX-2 inhibitors in invasive UCB.

Our study showed that high COX-2 immunostaining has a borderline statistically significant association with positive lymph node metastasis and tumors are associated with distant metastasis (*p* = 0.031). There are few studies that involved COX-2 immunostaining association with the metastatic potential of UCB. One study noted a significant correlation between COX-2 immunostaining and nodal metastasis [37]. On the other hand, another study did not show a significant correlation with lymph node metastasis [29].

In the current study, regression analysis revealed that high COX-2 immunostaining is an independent predictor of higher tumor grade, muscle invasion, advanced pathological T, lymphovascular invasion, and distant metastasis. A previous study reported that COX-2 correlated significantly with local invasion [24]. High COX-2 immunostaining may be used as an independent predictor of poor UCB outcome. Some suggested that the COX-2 expression may be associated with tumors of good prognosis [41]. However, in this study, the staining intensity was used to assess COX-2 immunostaining. In our study, high COX-2 immunostaining was associated with overall survival. However, there was no association with disease-free survival, a finding that had been reported previously [32], contrary to some previous studies that reported that high COX-2 immunostaining is associated with disease-specific survival [38, 41]. To sum up, the abovementioned contradictions in results of COX-2 immunostaining in UCB may be referred to as differences in sample sizes, cutoff points of COX-2 immunostaining, antibody clones, and immunostaining techniques.

## 5. Conclusion

In conclusion, the current study states that COX-2 immunostaining is associated with the invasiveness of UCB and supports that COX-2 immunostaining may be used as an independent prognostic biological marker in UCB. COX-2 may be a significant molecule in the initiation and progression of UCB. Subsequently, COX-2 inhibitors may be used as preventive and therapeutic agents in UCB. Molecular investigations and clinical trials are required to explore the molecular association of COX-2 expression with the development of UCB and effectiveness of COX-2 inhibitors as adjuvant therapy along with traditional chemotherapy.

## Figures and Tables

**Table 1:** Clinicopathological parameters of UCB (*n* = 123).

Parameter		Number (%)
Sex	Male	102 (82.9%)
	Female	21 (17.1%)

Age	<60 years	47 (38.2%)
	≥60 years	76 (61.8%)

Grade	Low grade	31 (25.2%)
	High grade	92 (74.8%)

Muscle invasion	Negative	42 (34.1%)
	Positive	77 (62.6%)
	Indeterminate	4 (3.3%)

Pathological stage (pT)	T1	46 (37.4%)
	T2	47 (38.2%)
	T3	17 (13.8%)
	T4	13 (10.6%)

Nodal metastasis	Negative	98 (79.7%)
	Positive	25 (20.3%)

Distant metastasis	Negative	110 (89.4%)
	Positive	13 (10.6%)

Lymphovascular invasion	Negative	101 (82.1%)
	Positive	22 (17.9%)

Anatomical stage	I	43 (35%)
	II	35 (28.5%)
	III	14 (11.4%)
	IV	31 (25.2%)

Local disease recurrence	Negative	83 (67.5%)
	Positive	40 (32.5%)

Survival	Alive	87 (70.7%)
	Dead	36 (29.3%)

T1: tumor invades subepithelial connective tissue; T2: tumor invades muscularis propria; T3: tumor invades perivesical tissue; T4: tumor invades any of the following: prostatic stroma, seminal vesicles, uterus, vagina, pelvic wall, or abdominal wall; stage I: T1, N0, and M0; stage II: T2, N0, and M0; stage III: T3 or T4a, N0, and M0; stage IV: any T and N1-3 or M1.

**Table 2:** Categories of COX-2 immunostaining in UCB and normal urothelium.

	Primary tumor (*n* = 123)	Normal urothelium (*n* = 25)	*p* value
Low immunostaining	102 (82.9%)	25 (100%)	0.033^∗∗^
High immunostaining	21 (17.1%)	0 (0%)	
*p* value	<0.001^∗^	<0.001^∗^	

^∗^One sample of the nonparametric chi-squared test; ^∗∗^Mann-Whitney *U* test.

**Table 3:** Correlation between COX-2 immunostaining and clinicopathological features of UCB.

Parameter		COX-2 immunostaining		*p* value
		Low	High	
Sex	Male	85	17	0.793^∗^
	Female	17	4	

Age	<60 years	41	6	0.320^∗^
	≥60 years	61	15	

Grade	Low grade	30	1	0.013^∗^
	High grade	72	20	

Muscle invasion	Negative	40	2	0.017^#^
	Positive	58	19	
	Indeterminate	4	0	

Pathological stage (pT)	pT1	44	2	0.003^#^
	pT2	58	19	
	pT3			
	pT4			

Nodal metastasis	Negative	85	13	0.08^∗^
	Positive	17	8	

Distant metastasis	Negative	100	10	0.031^∗^
	Positive	2	11	

Lymphovascular invasion	Negative	97	4	0.008^∗^
	Positive	5	17	

Anatomical stage	I	41	2	0.015^#^
	II	61	19	
	III			
	IV			

Local disease recurrence	Negative	69	14	0.931^∗^
	Positive	33	7	

Survival	Alive	71	16	0.548^∗^
	Dead	31	5	

^#^Kruskal-Wallis test; ^∗^Mann-Whitney *U* test; T1: tumor invades subepithelial connective tissue; T2: tumor invades muscularis propria; T3: tumor invades perivesical tissue; T4: tumor invades any of the following: prostatic stroma, seminal vesicles, uterus, vagina, pelvic wall, or abdominal wall; stage I: T1, N0, and M0; stage II: T2, N0, and M0; stage III: T3 or T4a, N0, and M0; stage IV: any T and N1-3 or M1.

**Table 4:** Regression analysis for COX-2 immunostaining in UCB.

Variable	Exp(*β*)	95% CI for exp(*β*)	*p* value
Grade	2.4	1.568-3.674	<0.001
Muscle invasion	0.153	0.034-0.692	0.015
Pathological stage (pT)	0.150	0.033-0.680	0.014
Lymphovascular invasion	0.259	0.091-0.736	0.011
Distant metastasis	0.272	0.079-0.938	0.039

## Data Availability

No data were used to support this study.

## Abbreviations

COX-2: Cyclooxygenase-2
UCB: Urothelial carcinoma of the urinary bladder.
